# Global Epidemiology of Tuberculosis and Progress Toward Meeting Global Targets — Worldwide, 2018

**DOI:** 10.15585/mmwr.mm6911a2

**Published:** 2020-03-20

**Authors:** Adam MacNeil, Philippe Glaziou, Charalambos Sismanidis, Anand Date, Susan Maloney, Katherine Floyd

**Affiliations:** ^1^Division of Global HIV and TB, Center for Global Health, CDC; ^2^Tuberculosis Monitoring and Evaluation, Global Tuberculosis Programme, World Health Organization.

Worldwide, tuberculosis (TB) is the leading cause of death from a single infectious disease agent (*1*), including among persons living with human immunodeficiency virus (HIV) infection (*2*). A World Health Organization (WHO) initiative, The End Tuberculosis Strategy, set ambitious targets for 2020–2035, including 20% reduction in TB incidence and 35% reduction in the absolute number of TB deaths by 2020 and 90% reduction in TB incidence and 95% reduction in TB deaths by 2035, compared with 2015 (*3*). This report evaluated global progress toward these targets based on data reported by WHO (*1*). Annual TB data routinely reported to WHO by 194 member states were used to estimate TB incidence and mortality overall and among persons with HIV infection, TB-preventive treatment (TPT) initiation, and drug-resistant TB for 2018 (*1*). In 2018, an estimated 10 million persons had incident TB, and 1.5 million TB-related deaths occurred, representing 2% and 5% declines from 2017, respectively. The number of persons with both incident and prevalent TB remained highest in the WHO South-East Asia and African regions. Decreases in the European region were on track to meet 2020 targets. Globally, among persons living with HIV, 862,000 incident TB cases occurred, and 1.8 million persons initiated TPT. Rifampicin-resistant or multidrug-resistant TB occurred among 3.4% of persons with new TB and 18% among persons who were previously treated for TB (overall, among 4.8% of persons with TB). The modest decreases in the number of persons with TB and the number of TB-related deaths were consistent with recent trends, and new and substantial progress was observed in increased TPT initiation among persons living with HIV. However, to meet the global targets for 2035, more intensive efforts are needed by public health partners to decrease TB incidence and deaths and increase the number of persons receiving TB curative and preventive treatment. Innovative approaches to case finding, scale-up of TB preventive treatment, use of newer TB treatment regimens, and prevention and control of HIV will contribute to decreasing TB.

TPT (the most common global regimen consists of daily isoniazid for ≥6 months) has been demonstrated to prevent TB disease among persons who might be infected with TB and are at risk for TB disease (*4*). Current WHO recommendations advise providing a course of TPT to all persons living with HIV and to all household contacts of persons with bacteriologically confirmed pulmonary TB disease (*5*).

TB data are reported to WHO annually by 194 member states and are reviewed and validated in collaboration with reporting entities (*1*,*6*). For each country, 2018 disease incidence (per 100,000 HIV-negative persons and per 100 persons living with HIV) was estimated from 1) TB prevalence surveys; 2) notifications from country surveillance systems adjusted by a standard factor to account for underreporting, overdiagnosis, and underdiagnosis; 3) national inventory studies that measure the level of underreporting of detected persons with TB combined with capture-recapture modeling; and 4) national notification data supplemented with expert opinion about case-detection gaps. Among HIV-negative persons, TB mortality rate estimates were based on all-cause mortality data from civil registration and vital statistics, mortality surveys, or the product of TB incidence and case fatality rate (CFR) (*1*). Among persons living with HIV, TB mortality rates were derived from the product of incidence and CFR. Data on persons receiving TPT represent numbers directly reported to WHO.

In 2018, an estimated 10 million persons had incident TB (132 per 100,000 population), a 2% decline from 2017 (Figure 1). Incidence has declined by an average of 1.6% per year since 2000. In 2018, 7.0 million persons globally were notified of their TB-positive results, representing 70% of the estimated number of persons with incident TB, an increase from the 6.4 million persons (64%) notified in 2017. In 2018, 69% of all persons with incident TB received anti-TB treatment, compared with 64% in 2017. The estimated number of TB-related deaths declined 5%, from 1.57 million in 2017 to 1.49 million in 2018 (CFR = 15%) (Figure 2). An estimated 862,000 persons living with HIV had incident TB in 2018, accounting for 8.6% of all persons with TB. Within this group, the estimated annual TB incidences were 6% in 2000, 2.5% in 2017, and 2.3% in 2018. In 2018, an estimated 251,000 TB deaths among persons living with HIV occurred (CFR = 29%). Overall, an estimated 484,000 persons had incident rifampicin-resistant or multidrug–resistant TB in 2018, representing 4.8% of all persons with TB, 3.4% of persons with a new TB diagnosis, and 18% of persons previously treated for TB. An estimated 214,000 persons died of either rifampicin-resistant or multidrug–resistant TB (CFR = 44%) in 2018. Among persons with rifampicin-resistant TB, 78% were estimated to have multidrug–resistant TB.

**FIGURE 1:**
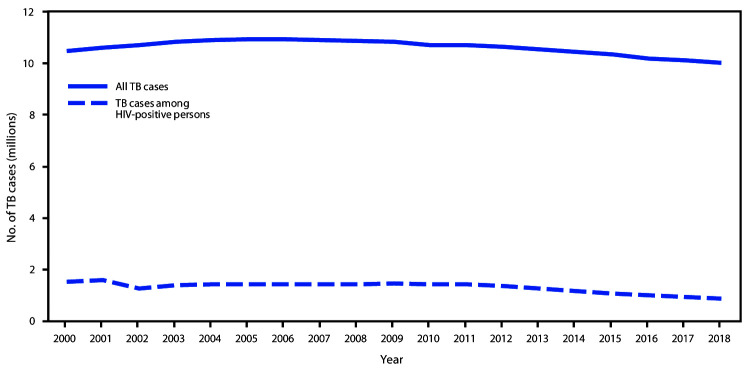
Trends in estimated incident tuberculosis (TB) among all persons and among persons living with human immunodeficiency virus (HIV-positive persons) — worldwide, 2000–2018 **Source:** Adapted with permission from World Health Organization. Global tuberculosis report 2019. Geneva, Switzerland: World Health Organization; 2019.

**FIGURE 2:**
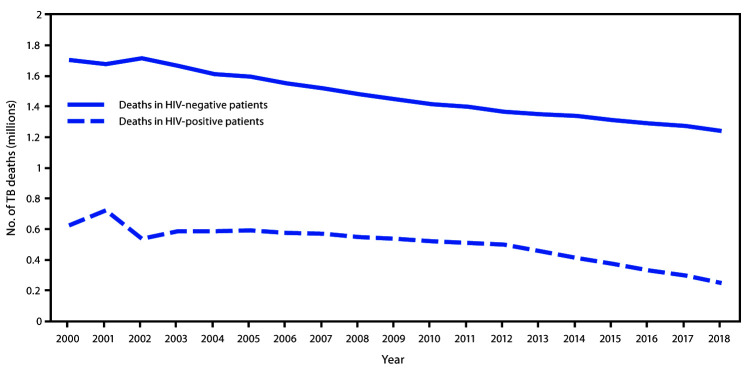
Trends in the estimated number of tuberculosis (TB)-related deaths among persons living with human immunodeficiency virus (HIV-positive persons) and HIV-negative persons — worldwide, 2000–2018 **Source:** Adapted with permission from World Health Organization. Global tuberculosis report 2019. Geneva, Switzerland: World Health Organization; 2019.

The WHO region of South-East Asia accounted for the highest percentage of TB cases (44% of all persons with TB) in 2018 (TB incidence = 220 per 100,000 population) (Table). TB incidence also was high in the African region (231 per 100,000 population) and, in 2018, this region accounted for 71% of all persons living with HIV with TB worldwide, similar to 2017. In the European region, TB incidence declined 15% since 2015 to 28 per 100,000 population. However, the proportion of persons with rifampicin-resistant or multidrug–resistant TB in this region (30%) remained substantially higher than that in all other regions (range = 3.1%–5.4%), and overall incidence of rifampicin-resistant or multidrug–resistant TB is similar to the Africa and South-East Asia regions.

**TABLE:** Estimated number of incident tuberculosis (TB) cases, TB incidence, and number of TB-associated deaths among all persons and persons living with human immunodeficiency virus (HIV-positive persons) and number of TB patients with rifampicin-resistant TB, by World Health Organization (WHO) region — Worldwide, 2018

WHO region	No. of persons with TB (x 1,000)	Incidence*	No. of deaths (x 1,000) (%)	No. of TB cases among HIV-positive persons (x 1,000)	No. of TB deaths among HIV-positive persons (x 1,000) (%)	No. rifampicin-resistant TB cases (x 1,000)	Incidence of rifampicin-resistant TB^†^	% of TB cases rifampicin-resistant^†^
**Global (all regions)**	**10,000**	**132**	**1,493 (15)**	**862**	**251 (29)**	**484**	**9.3**	**4.8**
African	2,450	231	609 (25)	615	211 (34)	77	7.3	3.1
Americas	289	29	23 (8)	29	5.9 (20)	11	1.0	3.8
Eastern Mediterranean	810	115	79 (10)	7	2.2 (32)	38	5.5	4.7
Europe	259	28	27 (10)	30	4.4 (15)	77	8.3	29.7
South-East Asia	4,370	220	658 (15)	140	21 (15)	182	9.2	4.1
Western Pacific	1,840	96	97 (5)	41	6.5 (16)	99	5.2	5.4

**Source:** Adapted with permission from World Health Organization. Global tuberculosis report 2019. Geneva, Switzerland: World Health Organization; 2019.

* Cases per 100,000 persons.

^†^ Includes multidrug-resistant TB.

In 2018, 65 countries reported data on TPT use among eligible persons living with HIV and 109 countries among children aged <5 years. Among these countries, 1.8 million persons living with HIV received TPT in 2018 (an 88% increase from 960,000 in 2017). Less progress was observed among eligible children aged <5 years: 350,000 children received TPT in 2018, a 20% increase compared with 292,000 in 2017.

## Discussion

WHO’s initiative, The End TB Strategy (*3*), has ambitious targets for 2020–2035, and the 2018 United Nations High Level Meeting on TB (UNHLM-TB) declaration established targets for 2022 that included providing TB treatment for 40 million persons infected with TB and providing TPT to 30 million persons, including 6 million persons living with HIV (*7*). Although some progress was made in 2018 toward meeting global targets, the overall number of persons with TB and TB-associated deaths decreased only slightly from 2017. Notable highlights in progress include an increased proportion of persons notified of TB-positive results, increased TPT among persons living with HIV, and decreased TB incidence in the European region.

The African region continues to have the highest HIV prevalence; thus, a large proportion of the TB cases in this region were associated with HIV. Similarly, the TB CFR among persons living with HIV continues to be high, and consequently, overall TB CFR was highest in this region. Whereas the European region is on track to meet 2020 targets, the overall proportion of persons with rifampicin-resistant or multidrug–resistant TB remains a substantial challenge. Recent progress in the development of new treatment regimens for TB and updated WHO guidelines suggest that all persons with rifampicin-resistant or multidrug–resistant TB could benefit from effective all-oral treatment regimens (*8*).

A key UNHLM-TB target is to initiate 30 million persons on TPT by 2022. Although the overall number of TPT initiations remains well below the target, including among household members of persons with TB, the number of persons living with HIV who initiated TPT nearly doubled from 2017 to 2018 and appears on track to meet the target of 6 million by 2022 (*1*). Substantial improvements in TPT initiation in other populations, including children aged <5 years who are household contacts of persons with TB, are necessary to reach the UNHLM-TB targets. Although daily isoniazid has been the primary TPT regimen globally, an alternative regimen of a 12-dose, once-weekly combination of isoniazid and rifapentine has been demonstrated to have similar efficacy with lower toxicity (*4*) and is anticipated to be increasingly used as a TPT regimen.

The findings in this report are subject to at least two limitations. First underlying data quality, particularly for surveillance, might affect the accuracy of estimates. Second, the differing methodologies used to generate country-level estimates might affect the comparability of estimates among regions and countries.

Global targets to end TB represent ambitious goals; however, achieving them will result in the prevention of disease and death among millions of persons. Although progress continues to be made, at the current pace of progress it remains unlikely that 2035 targets will be met. The scale-up of TB disease surveillance, initiation of TPT among eligible persons, and effective treatment need to continue to improve. Much more intensive efforts to find, cure, and prevent all cases of TB are necessary to meet global targets and end the public health burden of TB.

**Summary**

**What is already known about this topic?**

Targets for reducing tuberculosis (TB) have been set in a World Health Organization (WHO) initiative, The End TB Strategy. Achieving these targets will require substantial annual reductions in the incidence of TB and the number of TB deaths.

**What is added by this report?**

In 2018, an estimated 10 million incident TB cases and 1.5 million TB deaths occurred, reductions of 2% and 5%, respectively, from 2017. TB epidemiology varied by WHO region.

**What are the implications for public health practice?**

Innovative approaches to case finding, scale-up of TB preventive treatment, use of newer TB treatment regimens, and prevention and control of HIV will contribute to decreasing TB incidence.

## References

[1] World Health Organization. Global tuberculosis report 2019. Geneva, Switzerland: World Health Organization; 2019. https://www.who.int/tb/publications/global_report/en/

[2] Gupta RK, Lucas SB, Fielding KL, Lawn SD. Prevalence of tuberculosis in post-mortem studies of HIV-infected adults and children in resource-limited settings: a systematic review and meta-analysis. AIDS 2015;29:1987–2002. 10.1097/QAD.0000000000000802 26266773 PMC4568896

[3] World Health Organization. The End TB Strategy. Geneva, Switzerland: World Health Organization; 2015. https://www.who.int/tb/strategy/end-tb/en/

[4] Badje A, Moh R, Gabillard D, ; Temprano ANRS 12136 Study Group. Effect of isoniazid preventive therapy on risk of death in west African, HIV-infected adults with high CD4 cell counts: long-term follow-up of the Temprano ANRS 12136 trial. Lancet Glob Health 2017;5:e1080–9. 10.1016/S2214-109X(17)30372-8 29025631

[5] World Health Organization. Latent TB infection: updated and consolidated guidelines for programmatic management. Geneva, Switzerland: World Health Organization; 2018. https://www.who.int/tb/publications/2018/latent-tuberculosis-infection/en/30277688

[6] MacNeil A, Glaziou P, Sismanidis C, Maloney S, Floyd K. Global epidemiology of tuberculosis and progress toward achieving global targets—2017. MMWR Morb Mortal Wkly Rep 2019;68:263–6. 10.15585/mmwr.mm6811a3 30897077 PMC6478060

[7] Stop TB Partnership. UNHLM on TB: key targets and commitments. Geneva, Switzerland: STOP TB Partnership; 2020. http://www.stoptb.org/global/advocacy/unhlm_targets.asp

[8] World Health Organization. Rapid communication: key changes to the treatment of drug-resistant tuberculosis. Geneva, Switzerland; World Health Organization; 2019. https://www.who.int/tb/publications/2019/rapid_communications_MDR/en/

